# Cross-Talk between Transcriptome Analysis and Dynamic Changes of Carbohydrates Identifies Stage-Specific Genes during the Flower Bud Differentiation Process of Chinese Cherry (*Prunus pseudocerasus* L.)

**DOI:** 10.3390/ijms232415562

**Published:** 2022-12-08

**Authors:** Chunqiong Shang, Xuejiao Cao, Tian Tian, Qiandong Hou, Zhuang Wen, Guang Qiao, Xiaopeng Wen

**Affiliations:** 1 Key Laboratory of Plant Resource Conservation and Germplasm Innovation in Mountainous Region (Ministry of Education), Institute of Agro-Bioengineering, Guizhou University, Guiyang 550025, China; 2 Institute for Forest Resources & Environment of Guizhou, College of Forestry, Guizhou University, Guiyang 550025, China

**Keywords:** flower bud differentiation, carbohydrate, stage-specific genes, RNA-seq, *Prunus pseudocerasus*

## Abstract

Flower bud differentiation is crucial to reproductive success in plants. In the present study, RNA-Seq and nutrients quantification were used to identify the stage-specific genes for flower bud differentiation with buds which characterize the marked change during flower bud formation from a widely grown Chinese cherry (*Prunus pseudocerasus* L.) cultivar ‘Manaohong’. A KEGG enrichment analysis revealed that the sugar metabolism pathways dynamically changed. The gradually decreasing trend in the contents of total sugar, soluble sugar and protein implies that the differentiation was an energy-consuming process. Changes in the contents of D-glucose and sorbitol were conformed with the gene expression trends of *bglX* and *SORD*, respectively, which at least partially reflects a key role of the two substances in the transition from physiological to morphological differentiation. Further, the *WRKY* and *SBP* families were also significantly differentially expressed during the vegetative-to-reproductive transition. In addition, floral meristem identity genes, e.g., *AP1*, *AP3*, *PI*, *AGL6*, *SEP1*, *LFY,* and *UFO* demonstrate involvement in the specification of the petal and stamen primordia, and *FPF1* might promote the onset of morphological differentiation. Conclusively, the available evidence justifies the involvement of sugar metabolism in the flower bud differentiation of Chinese cherry, and the uncovered candidate genes are beneficial to further elucidate flower bud differentiation in cherries.

## 1. Introduction

Chinese cherry (*Prunus pseudocerasus* L.) belonging to the genus *Prunus*, in the Rosaceae family, is a commercially important fruit crop with high economic and ornamental values in China [1]. As a traditional fresh fruit, the Chinese cherry is rich in nutrients and trace elements that are suitable for a healthy diet [2]. Nowadays, the Chinese cherry industry is also thriving in its distribution areas due to its appealing sophistication.

Flower bud differentiation is one of the vital events in the life cycle of fruit trees. It is a physiological and morphological marker of the transition from vegetative growth to reproductive development [3]. The flower bud formation in the fruit tree consists of several consecutive phases such as: the induction of flower bud differentiation, histological transformation, and morphological differentiation [4]. The flower bud differentiation of sweet cherry may be roughly subdivided into seven stages based on paraffin sectioning, i.e., the physiological differentiation stage, the differentiation initiation stage, the floral stage, the sepal stage, the petal stage, the stamen stage, and the pistil primordia differentiation stages [5].

The differentiation of flower buds from induction to dormancy is influenced by diverse factors which are responsible for the cytochemical changes as well as hormone alterations [6]. The intrinsic factors that regulate flower bud differentiation are generally regarded as being the C/N ratio, the phytohormones, as well as the involvement of the florigen genes [7,8]. When the ratio of carbohydrates to available nitrogen compounds in the plants is high, it is conducive to reproductive growth and may promote bud differentiation; otherwise, it is conducive to vegetative growth and inhibits flowering [9]. The dynamic accumulation of sugars and nitrogen during flowering is significant, and when sugars are accumulated to a suitable amount, the nitrogen is conversed into the proteins needed for flowering [7]. Previous studies have demonstrated that the higher contents of soluble sugar and sucrose in the buds were beneficial to flower bud differentiation [6], and sucrose was also the initiation signals in flower induction [10]. Moreover, glucose is associated with early organ growth, contributing to the osmotic expansion of neighboring divided cells [11]. Specific amounts of amino acids and proteins are necessary for cell multiplication and morphogenesis to occur, thereby playing important roles in the process of flower bud differentiation [7]. Changes in nutrient signaling stimulate phytohormone signaling, and the synergistic action of the two nutrients may regulate plant growth and development. Several clearly defined types of plant hormones have been recognized, including gibberellin (GA), auxin, cytokinin (CK), and abscisic acid (ABA) [12], which may regulate the expression of corresponding genes responding to flower bud differentiation [13].

With the development of transcriptomic data, the genes and transcription factors involved in flower bud differentiation have been unraveled. Molecular and genetic studies of the annual model species *Arabidopsis* have shown that there is a complex genetic network which works to coordinate flowering that integrates six flowering pathways, i.e., the aging, autonomous regulation, vernalization, gibberellin, photoperiod, and ambient temperature pathways [14]. The first three pathways occur in a floral meristem, implying that there is a small range of central flowering regulators, e.g., transcription factors, including *SPLs* (*Squamosa promoter binding proteins*), *AGLs* (*Agamous-like protein*), *AP1* (*Apetala 1*), *LFY* (*Leafy*), *FT* (*Flowering locus T*), and *FD* (*Flowering locus D*), etc. Among them, *AP1* and *AGLs* belong to the *MADS* family, and together with *AP3* (*Apetala 3*), *PI* (*Pistillata*) and *SEP* (*Sepallata*) of the same family, and *AP2* (*Apetala 2*) which belongs to the *AP2/ERF* family, these genes are known as floral organ identity genes [15,16]. They work together as transcriptional complexes (ABCE model) to ensure the genes function to subtly express in each whorl of the developing flower [17]. Additionally, transcription factors such as bHLH, WRKY, NAC, and MYB are also the important regulatory proteins in flower bud differentiation [13].

The Chinese cherry is widely grown in South China [18]. The floral initiation and flower bud differentiation are highly stricken by the frequent occurrence of heavy rainfall in the summer, leading to a severe loss of yield [19]. Flower bud differentiation is a key developmental stage that directly affects the yield and quality of the Chinese cherry. To better understand the molecular regulatory mechanisms and the role of carbohydrates in flower bud differentiation of Chinese cherries, the objectives of the present investigation were: (1) to define the nodes and characteristics of each stage of flower bud differentiation; (2) to identify the ‘stage-specific’ genes involving in the stage regulation of flower bud differentiation; as well as (3) to reveal the roles of carbohydrates in the flower bud differentiation of Chinese cherries, particularly in heavy rainfall regions.

## 2. Results

### 2.1. Morphological Changes during Flower Bud Differentiation

**Figure 1:**
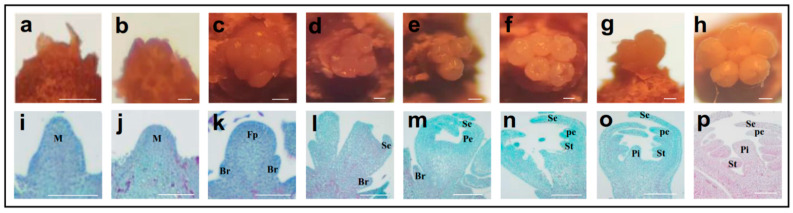
The anatomical and histological characterization in flower bud differentiation of *P. pseudocerasus* ‘Manaohong’. (**a**–**h**): internal morphology of individual flower bud observed using the stereomicroscope. (**i**–**p**): histological changes in individual flower bud. (**a**,**i**): physiological differentiation stage, undifferentiated meristematic apex is cone shaped; (**b**,**j**): differentiation initiation stage, the growth center begins to expand, and it is dome shaped; (**c**,**k**): floral primordium differentiation stage, from 2 to 6 small protrusions appeared; (**d**,**l**): sepal primordium differentiation stage, the growth center is flat, the surrounding parts are protruding, the whole of it is like a cup; (**e**,**m**): petal primordium differentiation stage, a new round of protrusions is generated on the inner side of the elongated sepal primordia; (**f**,**n**): stamen primordium differentiation stage, multiple protrusions are generated inside the elongated petal primordia; (**g**,**o**): pistil primordium differentiation stage, a new protuberance on the flat central part of the floral primordium; (**h**,**p**): differentiation has been completed. M: meristem; Br: bract primordium; Fp: floral primordium; Se: sepal primordium; Pe: petal primordium; St: stamen primordium; Pi: pistil primordium. Bars = 100 μm.

The flower bud differentiation of ‘Manaohong’ were classified into seven developmental stages, i.e., physiological differentiation, differentiation initiation, floral primordium, sepal primordium, petal primordium, stamen primordium, and pistil primordium (Figure 1; Table 1). From May to September, the development of the flower buds was observed in both rain-sheltered and shelter-free cultivation models, and the obvious differences in the differentiation stages between them were investigated. The physiological differentiation was confirmed by an undifferentiated meristematic apex with a cone-like shape around on 10 May, and this was termed as stage1 (Figure 1a,i). Then, it started to morphologically differentiate from early June (Figure 1b,j; Table 1). Next, the meristematic apex appeared as a dome-like structure with 2–6 floral primordia (Figure 1c,k), and then the sepal primordium was differentiated from the margin of dome-shaped apex, and the apex became cup shaped in stage3 (Figure 1d,l). In late July, the floral whorls continued their development, and the petal and stamen primordia were evidenced (Figure 1e,f,m,n; Table 1). In late August, the pistil primordium of most of the flower buds was observed (Figure 1g,o; Table 1). In late September, the flower bud differentiation was completed (Figure 1h,p). Based on the anatomical and histological characterization of flower bud differentiation, the buds of five stages (stage1–stage5) were sampled for the RNA-seq study of the floral transition.

### 2.2. Global Analysis of RNA-Seq Data

To identify the transcriptome changes of the floral transition in Chinese cherries, the buds of five differentiation stages (stage1–stage5) from both the shelter-free (abbreviated as ‘C’) and the rain-sheltered (abbreviated as ‘R’) patterns of ‘Manaohong’ were sampled for RNA-seq with us performing three biological replicates for each stage. A sum of 229.4 Gb of clean data was obtained, the clean data of each sample totaled more than 6.44 Gb, and the percentage of Q30 bases was above 92.97% (Table S1). The clean data of the mixed sample (blended from 30 samples) were de novo assembled into a reference transcriptome using Trinity software, and the assembly results were optimized for the evaluation. In total, 59,467 unigenes were produced (Table S2). The clean reads of each sample were compared with the reference sequence assembly to obtain the mapping results, and about 78.02% (76.10–79.58%) of the reads were mapped (Table S3).

### 2.3. Comparative Transcriptomic Analysis of Adjacent Differentiation Stages

**Figure 2:**
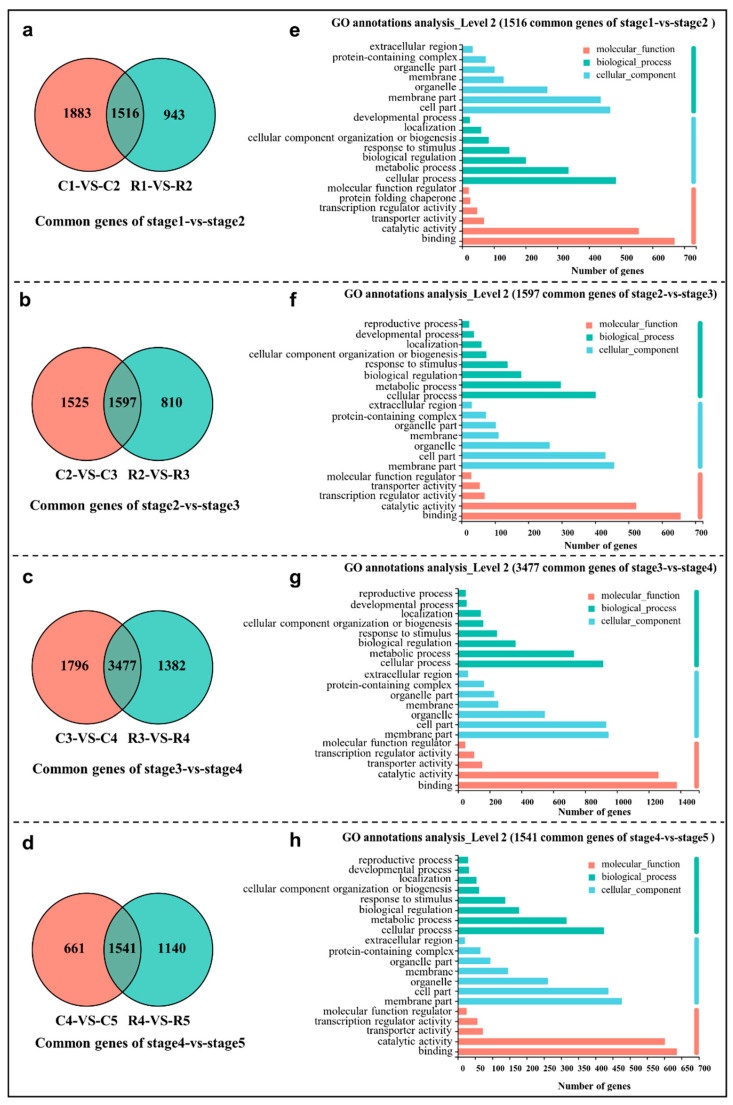
Venn diagram of common DEGs in stage1 vs stage2 (**a**), stage2 vs stage3 (**b**), stage3 vs stage4 (**c**), stage4 vs stage5 (**d**), and GO classification (**e**–**h**) of these common DEGs.

The differentially expressed genes (DEGs) in eight comparison groups (C1 vs C2, C2 vs C3, C3 vs C4, C4 vs C5; R1 vs R2, R2 vs R3, R3 vs R4, and R4 vs R5) were screened and collected using the combined criteria of |log_2_^FC^| ≥ 1 and an FDR < 0.05 (Figure S1). To uncover the DEGs that are differentiation stage dependent and independent of the grown pattern, four intersection analyses of two cultivation patterns were performed using a Venn diagram, i.e., C1 vs C2 and R1 vs R2, C2 vs C3 and R2 vs R3, C3 vs C4 and R3 vs R4, as well as C4 vs C5 and R4 vs R5 (Figure 2). In total, 1516, 1597, 3477, and 1541 common DEGs were obtained in the stage1 vs stage2 (Figure 2a), stage2 vs stage3 (Figure 2b), stage3 vs stage4 (Figure 2c), and stage4 vs stage5 analyses (Figure 2d), respectively. To further elucidate the functions of these common DEGs, a Gene Ontology (GO) classification analysis was carried out using the whole transcriptome as the background. In all of the four comparison groups, the ‘membrane part’, ‘cell part’, ‘organelle’, and ‘membrane’ were enriched in the cellular component (CC) category; the ‘cellular process’, ‘metabolic process’, ‘biological process’, ‘developmental process’, and ‘response to stimulus’ were enriched in the biological process (BP) category; ‘binding’, ‘catalytic activity’, ‘transporter activity’, and ‘transcription regulator activity’ were enriched in the molecular function (MF) category (Figure 2e–h).

### 2.4. Kyoto Encyclopedia of Genes and Genomes (KEGG) Pathway Analysis of Common DEGs at Four Comparison Groups

**Figure 3:**
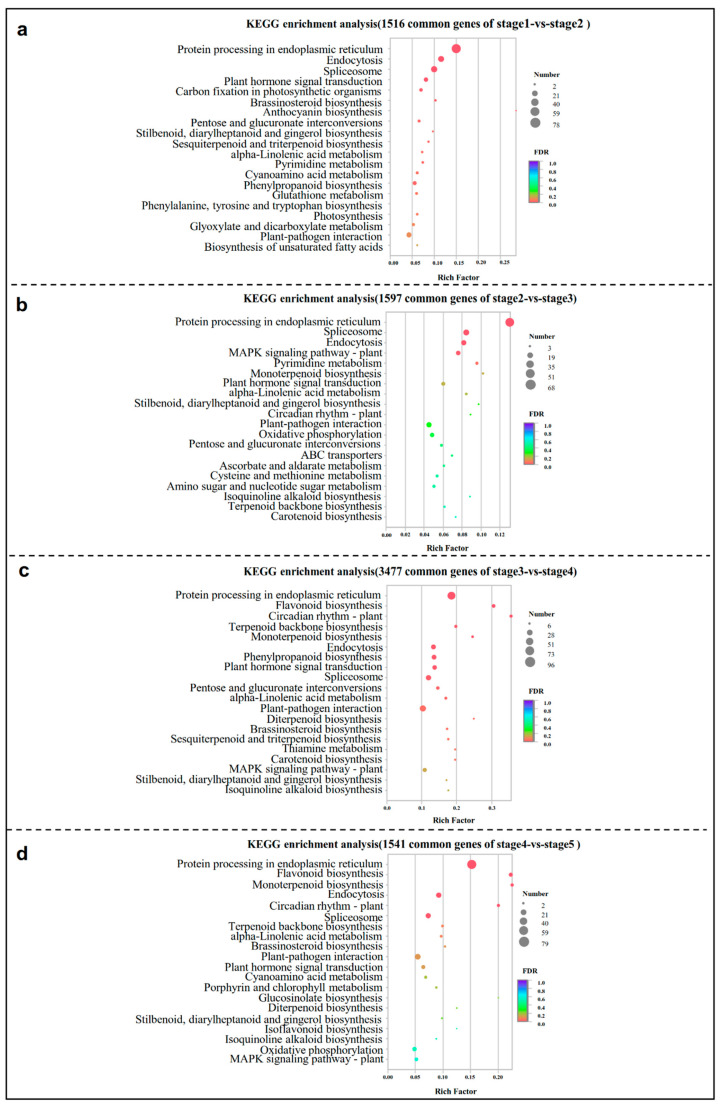
The 20 most enriched KEGG pathway of the common DEGs in stage1 vs stage2 (**a**), stage2 vs stage3 (**b**), stage3 vs stage4 (**c**), and stage4 vs stage5 (**d**). The rich factor represents the ratio of the number of DEGs involved in the pathway to the total number of annotated unigenes in this pathway.

The KEGG pathway mapping for 1516 common DEGs for stage1 vs stage2 was carried out, and the significantly (*p* < 0.05) enriched pathways included ‘Protein processing in the endoplasmic reticulum’, ‘Endocytosis’, ‘Spliceosome’, ‘Plant hormone signal transduction’, ‘Carbon fixation in photosynthetic organisms’, ‘Brassinosteroid biosynthesis’, ‘Anthocyanin biosynthesis’, ‘Pentose and glucuronate interconversions’, ‘Pyrimidine metabolism’, and other subcategories (Figure 3a). At stage2 vs stage3, ‘Protein processing in the endoplasmic reticulum’, ‘Endocytosis’, and ‘Spliceosome’ also ranked in the top three ones, which were followed by ‘MAPK signaling pathway’, ‘Pyrimidine metabolism’, ‘Monoterpenoid biosynthesis’, and ‘Plant hormone signal transduction’ (Figure 3b). At stage3 vs stage4, the first pathway was still the ‘endoplasmic reticulum’, but ‘Flavonoid biosynthesis’, ‘Circadian rhythm—plant’, ‘Terpenoid backbone biosynthesis’, and ‘Monoterpenoid biosynthesis’ began to become significantly enriched, which were followed by ‘Plant hormone signal transduction’, ‘Pentose and glucuronate interconversions’, and ‘Carotenoid biosynthesis’ (Figure 3c). In the last comparison group (stage4 vs stage5), the common genes were enriched for protein processing in the ‘endoplasmic reticulum’, ‘flavonoid biosynthesis’, ‘monoterpenoid biosynthesis’, ‘endocytosis’, ‘circadian rhythm’, ‘terpenoid backbone biosynthesis’, and other subcategories (Figure 3d). In general, ‘plant hormone signal transduction’ runs through the whole process of flower bud differentiation. In the early stage of differentiation, it was mainly related to the process of genetic information, nucleic acid metabolism, and carbohydrate metabolism. In the later stage, it mainly involved in the secondary metabolites.

### 2.5. Expressions of Carbohydrate-Related Genes and Carbohydrate Contents in the Buds across Differentiation Stages

**Figure 4:**
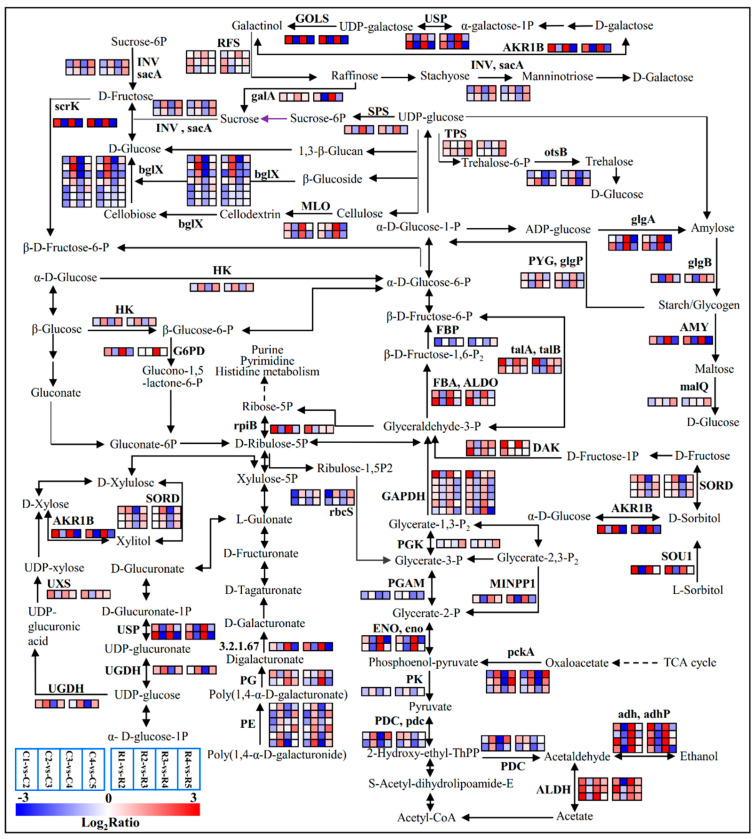
Significantly expressed genes involved in starch and sucrose metabolism, Glycolysis/Gluconeogenesis, the pentose phosphate pathway, Fructose and mannose metabolism, galactose metabolism, and the Pentose and glucuronate interconversions pathway during the differentiation phases of flower buds. Color codes from blue to red indicate changes in transcriptional expression levels between −3 and 3. The boxes from left to right indicate the expression values of a unigene obtained in C1 vs C2, C2 vs C3, C3 vs C4, C4 vs C5; R1 vs R2, R2 vs R3, R3 vs R4, R4 vs R5, respectively. Each row represents a separate unigene. This rule also applies in the next few pathways.

Most of the genes implicated in the sugar metabolism were differentially expressed during the process (Table S4 and Figure 4). Two *glgA* genes encoding starch synthase [EC:2.4.1.21] showed the trend of an ‘up—down–up–down’ pattern. Fructose-1,6-bisphosphatase [EC:3.1.3.11] gene *FBP* was down-regulated in the first and third comparison groups. The sucrose-phosphate synthase [EC:2.4.1.14] gene *SPS* was significantly up-regulated from stage3 to stage4. Seven unigenes encoding beta-glucosidase (bglX) [EC:3.2.1.21] related to D-glucose biosynthesis were down-regulated at stage1 vs stage2, up-regulated at stage2 vs stage3, and down-regulated at stage3 vs stage4. Sorbitol dehydrogenases (SORD) [EC:1.1.1.14] catalyze the reversible oxidation reduction of sorbitol, and three unigenes encoding SORD were up-regulated in stage2 vs stage3 and down-regulated from stage3 to stage4. The *UXS* (*UDP-glucuronic acid decarboxylase*) gene related to xylose synthesis was significantly up-regulated at the first and third comparison groups, but it was not significantly changed in the other two groups.

**Figure 5:**
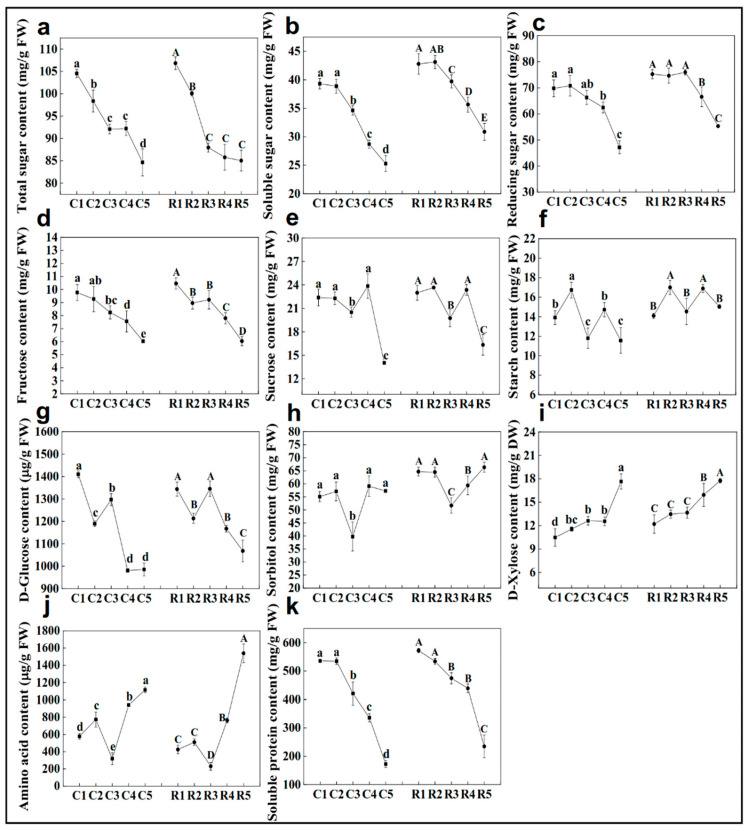
Changes of total sugar (**a**), soluble sugar (**b**), reducing sugar (**c**), fructose (**d**), sucrose (**e**), starch (**f**), D-glucose (**g**), sorbitol (**h**), D-xylose (**i**), amino acid (**j**), and soluble protein (**k**) concentrations during flower bud differentiation of Chinese cherries. C1–C5 represent the five stages of shelter-free cultivation, R1–R5 represent the five stages of rain-sheltered cultivation. Data are presented as the mean ± SD of three biological replicates. Different capital letters on the dots indicate significant differences between different stages of rain-sheltered model at *p* < 0.05 determined by Duncan’s tests, lowercase letters indicate significant differences of shelter-free model.

Variation in most of the carbohydrate concentration in the shelter-free and rain-sheltered models exhibited the same pattern. On the whole, the concentrations of total sugar, soluble sugar, and reducing sugar showed a trend of gradual decline (Figure 5a–c). The fructose content also showed a decreasing trend overall, except for in the stage3 of rain–shelter model (Figure 5d). The content of sucrose decreased significantly in stage3 and stage5 (Figure 5e). The starch content fluctuated throughout the development process (Figure 5f). The concentration of D-glucose was the highest at stage1, decreased at stage2, rose again at stage3, and then, it continued to decline (Figure 5g). The sorbitol content was higher than the starch, sucrose, and other carbohydrate ones were, and the content was the lowest at stage3 among the differentiation stages (Figure 5h). The D-xylose content demonstrated an upward trend on the whole during flower bud differentiation (Figure 5i). The content of amino acids presented a continuous upward trend except at stage3, where the content is the lowest (Figure 5j), conversely, the soluble proteins showed a gradual downward trend (Figure 5k).

### 2.6. Stage-Dependent transcriptional Activation and Repression Were Associated with Several Conserved Pathways

Several genes were significantly differentially expressed in the circadian rhythm-plant pathway (KO 04712), including *PRR5* (*two-component response regulator-like PRR5*), *PRR7* (*two-component response regulator-like PRR7*), *GI* (*Gigantea*), *ELF3* (*Early flowering 3*), *LHY* (*MYB-related transcription factor LHY*), *HY5* (*transcription factor HY5*), *FT*, *CO*, and *CHS* (*Chalcone and stilbene synthase*), most of which showed an up-regulated from stage3 to stage4 (i.e., C3 vs C4 and R3 vs R4), except for *HY5*, *LHY*, *CO,* and *CHS* (Figure S2, Table S5). The *FT* gene showed a progressive up-regulation throughout the differentiation process, however, *HY5* demonstrated progressive down-regulation. Several genes encoding CHS were down-regulated in the last two comparison groups.

**Figure 6:**
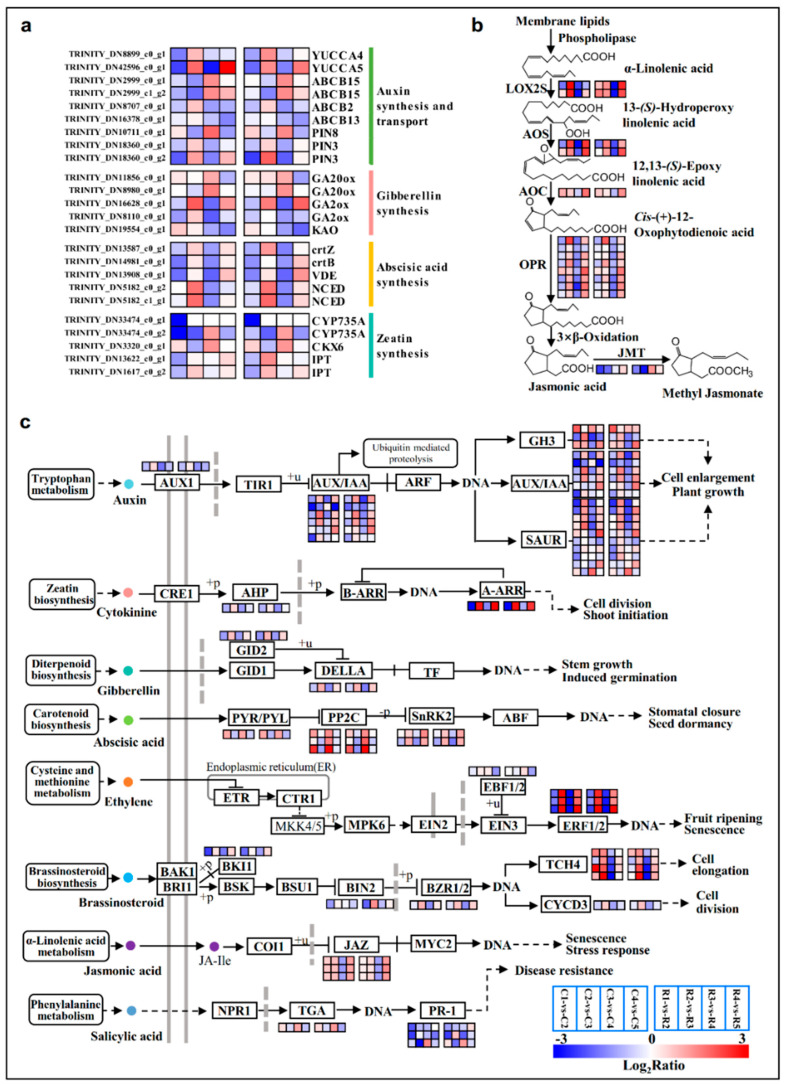
Significant expressed genes involved in IAA, ABA, GAs, Zeatin (**a**), and JA (**b**) biosynthesis during the differentiation phases, and the signal transduction pathways of these plant hormones (**c**). Color codes from blue to red indicate changes in transcriptional expression levels between −3 and 3. The boxes from left to right indicate the expression values of a unigene obtained in C1 vs C2, C2 vs C3, C3 vs C4, C4 vs C5; R1 vs R2, R2 vs R3, R3 vs R4, R4 vs R5, respectively. Each row represents a separate unigene.

The DEGs involved in phytohormone-related pathways were significantly modified during flower bud differentiation. YUCCA (Indole-3-pyruvate monooxygenase) is the most basic and important catalytic enzyme in the auxin synthesis pathway, currently; *YUCCA4* showed a down–up–down–up trend (Figure 6a and Figure S3o), and *YUCCA5* was significantly up-regulated at stage5 (Figure 6a; Table S6), and it had the highest expression at this stage (Figure S3p). Two key enzymes in the gibberellin synthesis pathway, *GA20ox* (Gibberellin 20 oxidase) and *GA2ox* (Gibberellin 2beta-dioxygenase), were significantly altered during floral bud differentiation. *GA20ox* was remarkably up-regulated in stage3 vs stage4, but the trend for *GA2ox* was opposite to this (Figure 6a; Table S6). Two genes encoding CYP735A (Cytokinin trans-hydroxylase) and two genes encoding IPT (Cytokinin synthase) associated with zeatin synthesis were highly down-regulated in stage1 vs stage2 (Figure 6a; Table S6). The majority of the genes encoding LOX2S (Lipoxygenase), AOS (Allene oxide synthase), AOC (Allene oxide cyclase), and OPR (12-oxophytodienoic acid reductase) enzymes in the jasmonic acid (JA) biosynthesis pathway exhibited persistent and significant changes throughout the differentiation progress (Figure 6b). The phytohormone signaling pathway integrates the signaling processes of multiple hormones including auxin, CK, GA, ABA, JA, ethylene, brassinosteroid, and salicylic acid (Figure 6c; Table S7). Most of the genes in the auxin signaling pathway demonstrated a trend of down–up–down–up, especially *AUX/IAA* (*Auxin/Indole acetic acid*) and *SAUR* (*SAUR family protein*) (Figure 6c; Table S7). During cytokinin and gibberellin signaling, *AHP* (*Histidine-containing phosphotransfer peotein*), *A-ARR* (*Two-component response regulator A-ARR family*), *GID2* (*F-box protein GID2*), and *DELLA* (*DELLA protein*) were significantly changed, and they showed the same trend (Figure 6c; Table S7). All of the genes of the abscisic acid signaling process were obviously differentially expressed, especially *PP2C* (*Protein phosphatase 2C*) and *SnRK2* (*Serine/threonine-protein kinase*), which showed opposite trends. Several *JAZ* (*Jasmonate ZIM domain-containing protein*) genes in the jasmonate signaling pathway were down-regulated in stage3 vs stage4, while they were up-regulated in the last group (Figure 6c; Table S7).

### 2.7. Major Transcription Factors Differentially Expressed during the Differentiation

**Figure 7:**
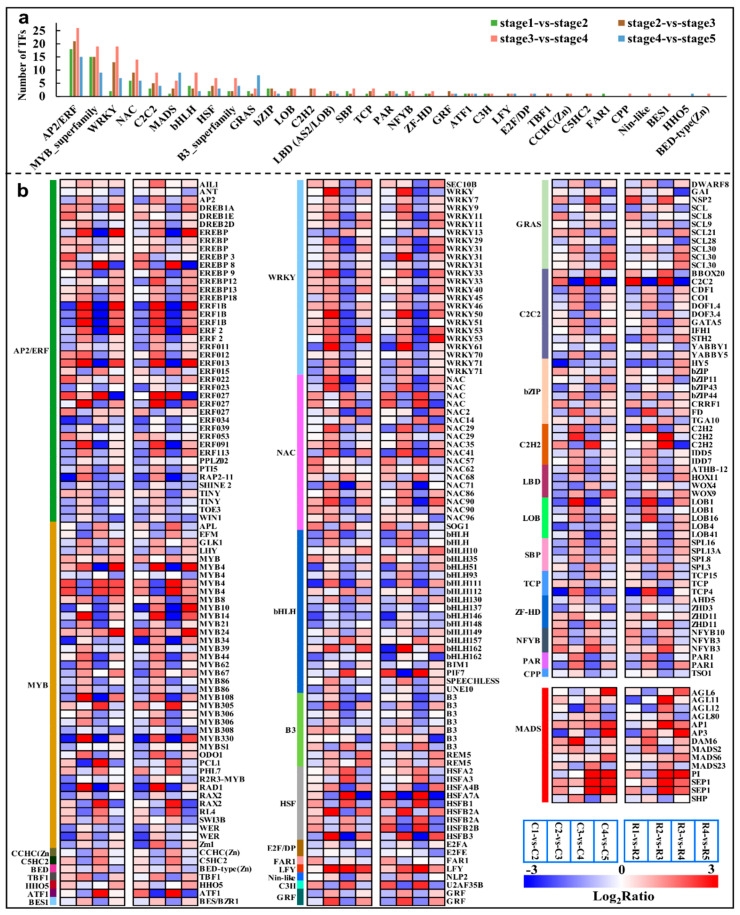
The types and numbers of TFs families in different comparison groups (**a**), and the relative expression of all identified TFs in either of the four comparison groups (**b**). Color codes from blue to red indicate changes in transcriptional expression levels between −3 and 3.

In the four comparison groups, a total of 252 common genes were identified as TFs that belonged to 33 gene families (Table S8). *AP2/ERF* (42, 16.7%), *MYB* (39, 15.5%), *WRKY* (24, 9.5%), *bHLH* (20, 7.9%), and *NAC* (19, 7.5%) were the most enriched gene families in all four of the comparison groups, which were followed by *MADS* (14, 5.6%) (Table S8 and Figure 7a). Furthermore, the highest number of transcription factors were annotated in stage2 vs stage3 and stage3 vs stage4, indicating a series of transcriptional changes from stage2 to stage4. In particular, changes in the expression of the *WRKY* family members were found to be especially pronounced. The vast majority of the members of the *WRKY* family were up-regulated in stage2 vs stage3 and down-regulated in stage3 vs stage4 (Table S8), e.g., *WRKY45*, *WRKY50*, *WRKY61,* and *WRKY71*. However, it is noteworthy that the transcript abundance of *WRKY13* has a completely opposite trend to that of the other members. These patterns revealed that most of the *WRKY* genes had a maximum transcript accumulation at stage3. So, most of *WRKYs* were appeared to be involved in the beginning of morphological differentiation in Chinese cherry flower buds, and they became good candidates for further analysis.

### 2.8. Expression Analysis of ABCE Model- and Flowering-Related Genes

**Figure 8:**
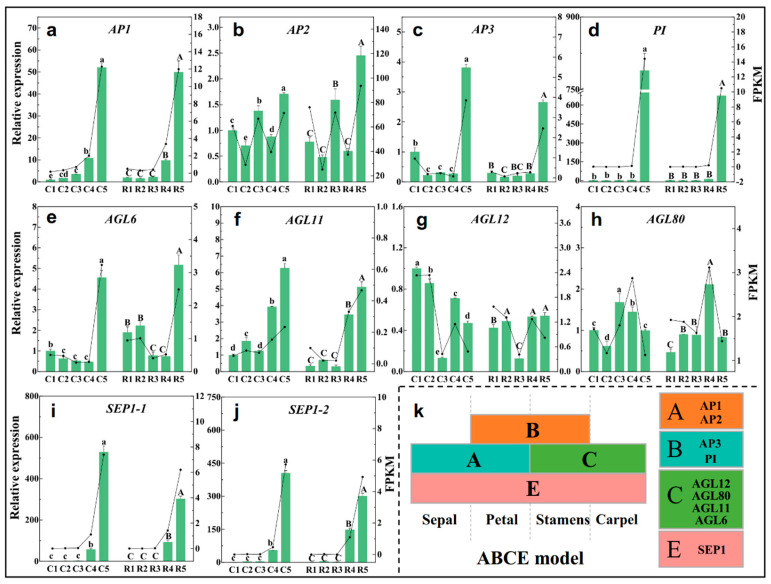
Expression of ABCE model genes at different differentiation stages in two cultivation patterns, *AP1* (**a**), *AP2* (**b**), *AP3* (**c**), *PI* (**d**), *AGL6* (**e**), *AGL11* (**f**), *AGL12* (**g**), *AGL80* (**h**), *SEP1-1* (**i**), *SEP1-2* (**j**), ABCE model (**k**). The histogram and left axis are the relative expressions measured by RT-qPCR, and the dot-line and right axis are the FPKM value obtained by RNA-Seq. C1–C5 represent the five stages of shelter-free cultivation, R1–R5 represent the five stages of rain-sheltered cultivation. Error bars show the Mean ± SD of the three biological replicates. Different capital letters on the bars indicate significant differences between different stages of rain-sheltered model at *p* < 0.05 determined by Duncan’s tests, lowercase letters indicate significant differences of shelter-free model.

The ABCE model of flower development proposes that the combined action of four sets of genes, the A, B, C, and E functional genes, designate the four floral organs (sepals, petals, stamens, and carpels, respectively) in a concentric floral whorl [20]. These four types of genes usually contain the MADS domains. Class A includes *AP1* and *AP2*, Class B consists of *AP3* and *PI*, Class C has *AGAMOUS*, and Class E has the *SEP* genes (Figure 8k). The RT-qPCR analysis showed that *AP1* expression was up-regulated continuously during the whole differentiation process (Figure 8a). The expression of *AP2* was significantly up-regulated at stage3 and stage5 (Figure 8b), but *AP3* and *PI* were only up-regulated at stage5 (Figure 8c,d). The Class C genes *AGL6* and *AGL11* were up-regulated at the later stages (Figure 8e,f), *AGL12* was down-regulated at stage3 (Figure 8g), but *AGL80* was up-regulated at stage3 or stage4 (Figure 8h). Two *SEP1* genes showed a similar expression pattern, and they were up-regulated from stage4, and the highest expression levels of them were observed at stage5 (Figure 8i,j).

In addition to the genes in the ABCE model, other genes or TFs involving in flowering were also verified by RT-qPCR in the present investigation. *TCP4* (*transcription factor TCP4*) had relatively high expression at stage1 and stage3 (Figure S3a), and *TCP9* had the highest expression at stage3 (Figure S3b). Five common DEGs were found to belong to the *SBP* (*Squamosa promoter-binding-like protein*) family in the four comparison groups (Figure 7), among which, *SPL8*, *SPL13A*, and *SPL16* had higher expression levels in stage1 and stage3 (Figure S3e–g), but the expression level of *SPL3* was the lowest in stage3 (Figure S3c). *SPL7* expression decreased continuously from stage1 to stage5 (Figure S3d). The expression of the *ELF4* (*Early flowering 4*) gene was higher in the latter two stages of development (Figure S3h), but the *FPF1* (*Flowering-promoting factor 1*) gene highly expressed in the first two stages (Figure S3i). The expression levels of *SOC1* (*Suppressor of overexpression of CO1*) and *CALA* (*Cauliflower A*) were the highest in stage1 (Figure S3j,k). *FD* expression showed an irregular pattern at each stage of flower bud differentiation (Figure S3l). *LFY* and *UFO* (*Unusual floral organs*) increased coincidently, and they were significantly up-regulated in the later stages of differentiation (Figure S3m,n).

### 2.9. RT-qPCR Validation of Differentially Expressed Genes (DEGs)

To validate the RNA-seq data, this study correlated the transcriptome results with the RT-qPCR data of the ABCE model genes and the differentiation-related genes mentioned above. The trend of the relative expression of most of the genes based on the RT-qPCR was consistent with the trend of the FPKM value of the RNA-Seq (Figure 8 and Figure S3), and the RT-qPCR results were highly correlated with the RNA-seq data (R^2^ = 0.9185) (Figure S4). Thereby, the transcript abundance estimation from the RNA-Seq is reliable.

## 3. Discussion

The flower bud differentiation of Chinese cherries ‘Manaohong’ was divided into seven stages: the physiological differentiation, differentiation initiation, floral primordium, sepal primordium, petal primordium, stamen primordium, and pistil primordium differentiation stages (Figure 1), which are similar to that which can be observed in sweet cherries [5]. To uncover the ‘stage-specific’ genes involving in stage-regulation of flower bud differentiation, stage samples of five time points were selected for the RNA-Seq, and many DEGs as well as pathways exhibited a stage-specific expression.

### 3.1. Roles of Carbohydrate- and Hormone-Related Genes in the Flower Bud Differentiation of Chinese Cherries

The KEGG enrichment results showed the DEGs were highly enriched in carbohydrate, nucleotide, and amino acid metabolism in the early stages of flower bud differentiation (Figure 3), suggesting that the energy and structural substances are essential for the initiation of differentiation, which was also documented in *Juglans sigillata* [7]. The synthesis of the carbohydrates and nucleic acids is the key to the transformation of the shoot apical meristem (SAM) from vegetative to reproductive growth [21], and the construction of a flower organ requires large amounts of these substances [22]. In the current case, the contents of the total sugar, soluble sugar, reducing sugar, and fructose were the highest at the physiological differentiation stage and the initiation stage (Figure 5), which further justifies that the buds should accumulate sugars at the physiological differentiation stage and then dissipate the sugars at the morphological differentiation stages. The content of D-glucose was the highest in the physiological differentiation stage, and it was decreased at the beginning of morphological differentiation, and the fluctuation trend was the same as the *bglX* genes’ expression, suggesting that a high glucose level was beneficial for morphological differentiation. Sorbitol is the primary photosynthate and translocated carbohydrate, most of which may be converted into fructose by sorbitol dehydrogenase (SORD) [23], and the content of it was also proved to be at a maximum early in the flower bud development process [24]. As we are currently aware, the sorbitol levels are higher in the physiological differentiation stage and the initiation stage. Then, the up-regulation of the *SORD* genes led to the decrease in the sorbitol content during the floral or sepal primordium differentiation stage. It indicated that the sorbitol was consumed to provide energy in the early stage of morphological differentiation of the flower bud in the Chinese cherry. In addition, sorbitol plays an important role in stamen development and pollen tube growth in apples, and the decreased sorbitol synthesis leads to abnormal stamen development [25]. Therefore, the increase in the sorbitol level in the last two stages favored stamen differentiation, and it also will facilitate an adaptation to freezing [26]. So, overall, the D-glucose and sorbitol were the key ‘stage-specific’ substances, and *bglX* as well as *SORD* were the ‘stage-specific’ genes that probably regulated the transition from physiological differentiation to morphological differentiation.

Endogenous hormones play important regulatory roles in all of the stages of flower development [27]. The *YUCCA*, *AUX/IAA*, and *SAUR* genes involving in auxin synthesis and signaling were down-regulated from the physiological differentiation stage to the initiation stage (Figure 6a,c). A previous study reported that the young organs with high concentrations of free IAA could inhibit or delay the initiation, and having a low IAA content in the early stage was beneficial to flower induction [12]. *GA2ox* and *GA20ox* are two key enzymes in GA biosynthesis. *GA2ox* regulates plant growth by inactivating endogenous bioactive GAs [28]. *GA20ox* is the opposite to this because it controls the biosynthesis of GA by oxidizing several precursors [14]. In the present case, *GA2ox* and *GA20ox* gave opposite expression trends (Figure 6a; Table S6). DELLA proteins are a class of nuclear growth inhibitors that promote the elongation of plant organs when DELLA is inactivated by GA [29]. So, the *GA2ox* and *DELLA* were down-regulated in stage3 vs stage4 (Figure 6a,c), and *GA20ox* being up-regulated may cause the accumulation of GA in this period, thereby promote the differentiation of petals and stamen [30]. Several genes, e.g., *crtZ* (*beta-carotene 3-hydroxylase*), *crtB* (*15-cis-phytoene synthase*), *VDE* (*Violaxanthin de-epoxidase*), *NCED1* (*9-cis-epoxycarotenoid dioxygenase 1*), and *NCED5,* which were involved in ABA biosynthesis, displayed similar patterns in their expression regulation, i.e., down–up–down–up (Figure 6a; Table S6). The fluctuation trend was proved to be similar to that of the ABA level during flower bud differentiation in other species [31,32]. The up-regulation of the final stage may be related to the favorable morphological differentiation of the flower buds due to high ABA levels, especially during the development of the pistil primordia [33]. JA may act as a signaling molecule to regulate sexual reproduction [34]. In this study, the ‘α-linolenic acid metabolism’ pathway associated with JA synthesis was highly enriched in all of the four comparison groups (Figure 3), the unigenes encoding key enzymes LOX2S, AOS, AOC, and OPR in this pathway (Figure 6b), and JAZ related to signaling (Figure 6c) were all obviously differentially expressed from sepal formation to the termination of differentiation, suggesting that JAs should regulate the formation of floral organs, especially filament elongation and pistil formation [35,36].

### 3.2. Involvements of WRKYs and SBPs in Regulating the Vegetative-to-Reproductive Transition of Flower Bud Differentiation

Transcription factors play an important role in regulating downstream targets, thus, they contribute highly to the biological functions in plant growth and development. In the current case, *AP2/ERF*, *MYB*, *WRKY*, *NAC*, and *bHLH* were differentially expressed in a high number of unigenes throughout flower bud differentiation (Figure 7), and these families also appeared in large numbers during the differentiation process of *P. avium* [5]. In total, 24 *WRKY* members were identified across the whole differentiation process (Table S8), most of which, except for *WRKY13*, were significantly up-regulated at the onset of morphological differentiation (Table S8), indicating the importance of *WRKYs* in the transition of morphological differentiation in the flower buds of the Chinese cherry. It has been shown that *WRKY71* is involved in the flower primordia differentiation of wintersweet [37], and *AtWRKY71* could accelerate flowering via the direct activation of *AP1*, *FT,* and *LFY* in *Arabidopsis* [38]. Therefore, based on the changes in its expression, *WRKY71* was presumed to be involved in the regulation of flower bud morphological differentiation and the flowering time in the Chinese cherry. In the same fashion, a study has shown that *WRKY45* was significantly expressed in capitulum at the flowering stages [39], which supported the up-regulation of the *WRKY45* gene at the floral stage in the current study (Table S8). In addition, the specificity of *WRKY13* in terms of its expression changes may be related to its negative regulation of the flowering time [40]. Accordingly, *WRKYs* probably are of high importance for the transition from physiological to morphological differentiation for flower buds in Chinese cherries, whose molecular roles should be further illuminated.

The *SQUAMOSA promoter binding protein-like* (*SPL*) gene family, which is an SBP-box transcription family, had comparatively fewer annotated members in this study, and they were also confirmed to play an important regulatory role in flower bud differentiation, including in the regulation of vegetative phase change, floral transformation, flowering time, and inflorescence development [41,42]. In current study, the *SPL* genes showed distinct spatiotemporal expression patterns. *SPL8*, *SPL13A,* and *SPL16* were highly up-regulated in the physiological differentiation stage (Figure S3e–g). Furthermore, the high expression levels of *SPL8*, *SPL13A*, and *SPL16* at the floral differentiation stage further confirmed the role of *SPLs* in regulating the vegetative-to-reproductive transition in flower bud differentiation and by different modulations [41,42]. *SPL3* did not play a major role in the vegetative phase change or flowering, but it promoted the floral meristem identity transition by activating the flower meristem-specific genes, *LFY* and *AP1* [43]. *SPL7* and *SPL8* induced phase transition and flowering in grasses by directly upregulating *SEP3* (*SEPALLATA3*) and *MADS32* [44]. In addition, *SPL8* was found to be involving in GA signaling and positively regulated trichome formation on the sepals’ and stamens’ filament elongation [45,46]. In present study, *SPL8* was highly expressed at the sepal and stamen primordia differentiation stage (Figure S3e). These data suggested that *SPLs* have a role during floral organ primordium differentiation in Chinese cherries.

### 3.3. The Implication of Floral Meristem Identity Genes and Flowering-Time Genes in Flower Bud Differentiation

In the present study, 14 *MADS* family genes were identified (Figure 7). *AP1*, the *AGLs*, *PI,* and *SEP1* belong to the *MADS* family, and together with *AP2* (belonging to *AP2/ERF*), they are involved in the traditional ABCE floral organ model, and they were identified as floral meristem identity genes. In addition, *LFY* and *UFO* were also identified as floral meristem identity genes [47,48]. These floral meristem identity genes were activated to play a pivotal role in specifying the floral meristems during floral transition [49]. *UFO*, together with *LFY*, positively regulated the class B organ function by activating *AP3* and *PI* transcription, respectively [50,51,52]. Furthermore, LFY might directly act on the promoter of *AP1* and *SEP*, activating their expression [53,54,55]. Additionally, these genes were also proved to be involved in sepal and petal identity specification as well as bract suppression [47,56,57,58]. In present study, *UFO*, *LFY*, *AP1*, *AP3*, *PI,* and *SEP1* showed a same trend, and they were significantly up-regulated at the petal and stamen primordia differentiation stages (Figure 8 and Figure S3), which further confirms that they are required for petal and stamen primordia identification in Chinese cherries. *CAL* and *AP1* are a pair of paralogous genes; *AP1* played a major role in specifying the floral meristem identity in a largely redundant fashion with *CAL* [59]. In present study, the opposite expression patterns of *CAL* and *AP1* suggests that *CAL* may play a role in physiological differentiation, while *AP1* plays a role mainly in petal and stamen primordia identity. In several species, *AGL6* performs *SEP-like* functions [60,61,62]. Thus, in the current study, *AGL6,* which is similar to *SEP1*, was also involved in the specification of the petal and stamen primordia identity of Chinese cherry flower bud differentiation.

In addition to the floral meristem identity genes mentioned above, the flowering time gene was also identified. *FPF1* is among the earliest expressed genes that are induced specifically in the apical meristem after floral induction [63]. It modulates the competence for flowering of apical meristems, and the overexpression of *FPF1* would initiate the earlier formation of abaxial trichomes [64]. So, the high expression of *FPF1* during the physiological differentiation and initiation differentiation stages of the Chinese cherry flower bud was beneficial in promoting the onset of morphological differentiation. Overall, our results provided a better understanding for the functional roles of the key genes such as *UFO*, *LFY*, and *FPF1* involved in the flower bud differentiation of Chinese cherries.

## 4. Materials and Methods

### 4.1. Plant Materials and Sample Collection

The trial site was located in a public orchard in Fuquan city (26°70′ N; 107°51′ E) in central Guizhou Province, China. It has a subtropical monsoon climate with an annual average temperature of 14 °C and relative humidity of 88%. The total annual precipitation is 1220 mm. Seven-year-old cherry plants (cultivar ‘Manaohong’) with similar growth potential were used as the test materials, at a spacing of 3 × 3 m^2^, and all of the plants were naturally pollinated. The rain–sheltered cultivation facilities are arched with steel pipes, the steel frame body is 30 m × 10 m × 4 m, and the steel frame is covered with high-transmittance (rate of approximately 70%) PE film. The rain shelter is semi-sheltered and ventilated on all of the sides. The previous year’s flower buds were all in full bloom on 28 February 2021, so the flower buds for next year were sampled between May and October 2021 on twelve sampling dates (10 May; 20 May; 30 May; 10 June; 20 June; 1 July; 15 July; 30 July; 15 August; 30 August; 15 September; 30 September). We randomly selected about 330 terminal buds from the top of the one-year-old shoots in four directions of ten trees under the sheltered and unsheltered conditions, of which, thirty flower buds were used as fixed specimens for the anatomical observation. The remaining three hundred buds were mixed and immediately frozen in liquid nitrogen and then stored at −80 °C for RNA isolation. Three biological repeats were performed. They were sampled every time at 11 a.m.

### 4.2. Morphological Analysis of the Flower Bud Transition

Each terminal bud was dissected and observed using a stereoscopic anatomical microscope (XTS20, Tech, Beijing, China) to estimate its differentiation stage. Thirty flower buds were fixed in FAA (5% formalin, 45% ethanol, 5% glacial acetic acid, and 45% water, *v*/*v*) for more than 24 h at 4 °C. The samples were dehydrated sequentially in 50%, 70%, 80%, and 95% ethanol for 1.5 h and twice in 100% ethanol at 0.5 h intervals. The reagent was replaced with a solution of xylene and ethanol (1:1, *v*/*v*) for 12 h, which was followed with xylene, first for 1.5 h, then, in fresh xylene for 1.5 h. All of the procedures were carried out at room temperature (26 ± 2 °C). The samples were decolorized in a mixture of xylene and paraffin (1:1, *v*/*v*) for 10 h, which was a procedure that was followed three times in 100% paraffin at 3 h intervals, and all of these were performed at 60 °C. Then, the flower buds were embedded in paraffin. The paraffin-embedded samples were sectioned to 10 mm thickness using a microtome (YD-315, YiDi, Jinhua, China). The sections were sequentially washed with ethylene glycol ether acetate for 37 °C for6 h and 37 °C for12 h, and they were left at room temperature for 10–15 min. Then, we soaked the sections in 100% ethanol for 10 min and changed the reagent twice, and the samples were stripped of the reagents in a 100%, 95%, 90%, and 80% ethanol series for 10 min. Next, we washed the sections with tap water. The slices were stained in safranin for 3–5 s and then washed to remove the excess dye. Then, the slices were placed in 50%, 70%, and 80% gradient alcohols for 3–8 s. Immediately after this, the slices were stained in fast green for 4–6 s and dehydrated three times in ethanol. Finally, the sections were placed in xylene for 5 min and sealed with neutral resin, and then, they were observed under a microscope (ECLIPSE Si, Nikon, Tokyo, Japan).

### 4.3. Determination of Carbohydrates, Amino Acids, and Soluble Protein

All of the indexes were determined by the kit of the same company (Beijing Slolarbio Science & Technology Co., Ltd., Beijing, China). The catalog numbers of the total sugar, soluble sugar, reducing sugar, fructose, sucrose, starch, sorbitol, and D-Xylose were BC2710, BC0030, BC0230, BC2450, BC2460, BC0700, BC2520, and BC4390, respectively. The concentration of the D-glucose content was determined by a glucose oxidase peroxidase (GOPOD) kit (catalog number: BC2500).

### 4.4. RNA Extraction, Library Construction, and Sequencing

The flower buds were collected at 5 dates (stage1: 10 May; stage2: 10 June; stage3: 1 July; stage4: 30 July; stage5: 30 August) from two cultivation patterns were used for the RNA-Seq. The shelter-free cultivation samples were abbreviated as ‘C’, and the rain-sheltered cultivation samples were abbreviated as ‘R’. The total RNA extraction was performed using a Plant RNA Purification Reagent (Invitrogen, Carlsbad, CA, USA). Three biological replicates were set up for each time point. There were a total of thirty samples. In addition, a mixed sample of five time point samples was used for the de novo RNA transcriptome analysis. The concentration and quality of the extracted RNA were calculated using a NanoDrop 2000 spectrophotometer (NanoDrop Thermo Scientific, Wilmington, DE, USA). The RIN value of the RNA was measured using Agilent 2100 (Agilent, Santa Clara, CA, USA), and the integrity of the RNA was verified by 1% agarose gel electrophoresis. The RNA with total RNA quantity ≥ 1 ug, concentration ≥ 35 ng/μL, OD260/280 ≥ 1.8, and OD260/230 ≥ 1.0 were selected for the library construction. The construction and sequencing of the cDNA libraries were performed by Shanghai Majorbio Bio-pharm Biotechnology Co., Ltd. (Shanghai, China) on an Illumina Hiseq Xten/NovaSeq 6000 (Illumina, San Diego, CA, USA) using a 2 × 150 bp paired-end sequencing method.

### 4.5. De Novo Assembly and Annotation

The acquired raw data were processed for quality control using Sickle (https://github.com/najoshi/sickle, accessed on 12 September 2021) and SeqPrep (https://github.com/jstjohn/SeqPrep, accessed on 13 September 2021). Then, the clean data from the mixed sample consisting of a little bit of each sample were used to perform the de novo assembly with Trinity (http://trinityrnaseq.sourceforge.net/, accessed on 15 September 2021) [65]. Using BLASTX, all of the assembled transcripts were searched in the NCBI protein non-redundant (NR), COG, and KEGG databases to find the proteins with the highest sequence similarity, and we performed the functional annotation on these proteins, and we set typical cutoff E-values of less than 1.0 × 10^−5^. The BLAST2GO (http://www.blast2go.com/b2ghome, accessed on 16 September 2021) [66] program was used to obtain the GO annotations of a unique assembly of transcripts to characterize the biological processes, cellular components, and molecular functions. The metabolic pathway analysis was performed using the Kyoto Encyclopedia of Genes and Genomes (KEGG, http://www.genome.jp/kegg/, accessed on 17 September 2021) [67].

### 4.6. Mapping, Differential Expression Analysis, and Functional Enrichment

The clean reads of each sample were compared with the reference sequence obtained by Trinity assembly to obtain the mapping results for each sample. The expressed level of each gene was calculated using RSEM software (Version 1.3.1) (http://deweylab.github.io/RSEM/, accessed on 18 September 2021). The differently expressed genes (DEGs) between the different comparison groups (C1 vs C2, C2 vs C3, C3 vs C4, C4 vs C5, R1 vs R2, R2 vs R3, R3 vs R4, and R4 vs R5) (where the latter was compared to the former) were determined using DEGseq2 algorithms (http://bioconductor.org/packages/stats/bioc/DESeq2/, accessed on 20 September 2021) with an expression difference threshold of Q value ≤ 0.05 and |log_2_^FC^| ≥ 1 and FDR < 0.05. The GO and KEGG identification was performed at a Bonferroni-corrected *p*-value ≤ 0.05, which was compared to the whole transcriptome background to determine in which GO terms and pathways DEGs were significantly enriched.

### 4.7. Validation of RNA-Seq Analysis by RT-qPCR

Twenty-six DEGs were selected to validate the RNA-Seq analysis data, including 10 ABCE model genes and 16 flowering genes, and the gene-specific fluorescent primers are shown in Table S9. The quantitative real-time PCR (RT-qPCR) was performed on a CFX Connect™ Real-Time PCR Detection System (Bio-Rad, Hercules, CA, USA). For each developmental period, the RNA samples of three biological replicates were used for the RT-qPCR validation. First-strand cDNA was generated using a PrimeScriptTM RT reagent kit using gDNA Eraser (Code No. RR047A, Takara, Tokyo, Japan) according to the manufacturer’s protocol. The fluorescence reactions were performed in 10 μL volumes containing SYBR Green Fast qPCR Mix (Biomarker, Beijing, China) and a quantity of cDNA corresponding to 6 ng of the total RNA. The relative expression was calculated according to the 2^−ΔΔCT^ algorithm using the *RSP* (*40S ribosomal protein*) gene as the internal reference gene [68], C1 as a reference sample, and the relative expression of each gene at C1 was set to 1.

### 4.8. Statistical Analysis

All of the data were statistically analyzed in SPSS 22.0 software (Chicago, IL, USA) using a one-way ANOVA. Duncan’s test was used to determine the statistically significant differences among the developmental stages. If the *p*-value was less than 0.05, then the mean difference between the time points was considered to be significant. The final rendered image was graphed using Origin 9.0 (Origin Lab, Northampton, MA, USA).

## 5. Conclusions

The present study integrates the histological and molecular information of the Chinese cherry to decipher the key features associated with flower bud differentiation. The decrease in the total sugar, reducing sugar, soluble sugar, and soluble protein levels indicates that flower bud differentiation is a process of gradual energy consumption. *bglX* as well as *SORD* were the ‘stage-specific’ genes regulating the morphological differentiation of flower buds in the Chinese cherry. By comparing the transcriptomes between the adjacent stages, several pathways, e.g., carbohydrate metabolism, the circadian rhythm, and plant hormone signal transduction, were justified to be associated with flower bud differentiation. The members of the *AP2/ERF*, *MYB*, *WRKY*, *NAC*, *bHLH*, and *MADS* families were the most represented among the DEGs, suggesting that their functions are in flower bud differentiation. Furthermore, the differential expression of the *SBP* family genes (e.g., *SPL3/7/8/13A/16*), the floral meristem identity genes (e.g., *AP1*, *AP2*, *AP3*, *PI*, *AGLs*, *SEP1, UFO,* and *LFY*), and the flowering time genes (e.g., *FPF1, ELF4,* and *FD*) were phase specific, indicating that their key roles are in initiation differentiation or organ primordium differentiation. The obtained data might serve as a valuable resource for further elucidating the molecular mechanisms of flower bud differentiation in Chinese cherries, and further validation of the genes will be the key emphasis of our future research.

## Figures and Tables

**Table 1:** Flower bud differentiation stages of ‘Manaohong’ and their percentage (%) in the observed buds.

Date (Month/Day)	Cultivation Patterns	Physiological Differentiation	Differentiation Initiation	Floral	Sepal	Petal	Stamen	Pistil
				Primordia Differentiation				
10 May (stage1)	C	100.0	-	-	-	-	-	-
	R	100.0	-	-	-	-	-	-
20 May	C	90.0	10.0	-	-	-	-	-
	R	93.3	6.7	-	-	-	-	-
30 May	C	46.7	53.3	-	-	-	-	-
	R	50.0	50.0	-	-	-	-	-
10 June (stage2)	C	16.7	83.3	-	-	-	-	-
	R	23.3	76.7	-	-	-	-	-
20 June	C	-	50.0	46.7	3.3	-	-	-
	R	-	53.3	46.7	-	-	-	-
1 July (stage3)	C	-	6.7	46.7	36.6	10.0	-	-
	R	-	20.0	43.4	33.3	3.3	-	-
15 July	C	-	-	16.7	53.3	30.0	-	-
	R	-	-	23.3	56.7	20.0	-	-
30 July (stage4)	C	-	-	-	10.0	56.7	30.0	3.3
	R	-	-	-	20.0	53.3	26.7	-
15 August	C	-	-	-	-	30	46.7	23.3
	R	-	-	-	-	36.7	43.3	20.0
30 August (stage5)	C	-	-	-	-	3.3	66.7	30.0
	R	-	-	-	-	10.0	63.3	26.7
15 September	C	-	-	-	-	-	16.7	83.3
	R	-	-	-	-	-	20.0	80.0
30 September	C	-	-	-	-	-	-	100.0
	R	-	-	-	-	-	-	100.0

Note: The values in each row indicate the percentage for the buds in corresponding stage to the total buds observed; R: Rain–shelter model; C: Shelter-free model. Flower buds were collected at five dates (stage1–stage5), and two growth pattern samples were used for subsequent carbohydrate content determination and RNA-Seq analysis.

## Data Availability

The NGS datasets mentioned in the manuscript are available on NCBI SRA database under project PRJNA903958.

## Supplementary Materials

The following supporting information can be downloaded at: https://www.mdpi.com/article/10.3390/ijms232415562/s1.
