# Supplementary material for: Cross-Talk between Transcriptome Analysis and Dynamic Changes of Carbohydrates Identifies Stage-Specific Genes during the Flower Bud Differentiation Process of Chinese Cherry (Prunus pseudocerasus L.)
Source: Int J Mol Sci. 2022 Dec 8;23(24):15562. doi: 10.3390/ijms232415562 (PMC9778666; doi:10.3390/ijms232415562)
Supplement: Supplementary file 1 [file ijms-23-15562-s001.zip › ijms-2009006-supplementary/supplementary/Supplementary Figures.pdf]

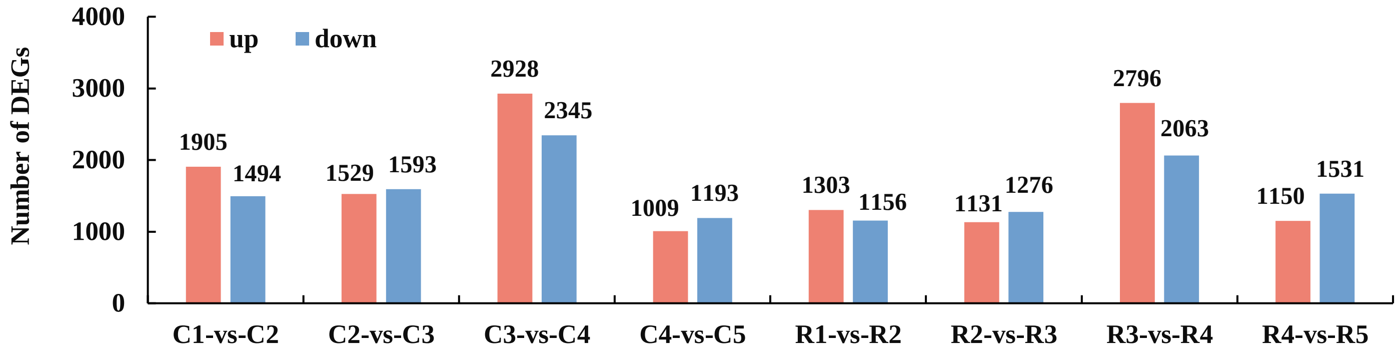

Figure S1 The number of differentially expressed genes (DEGs) between adjacent differentiation stages.

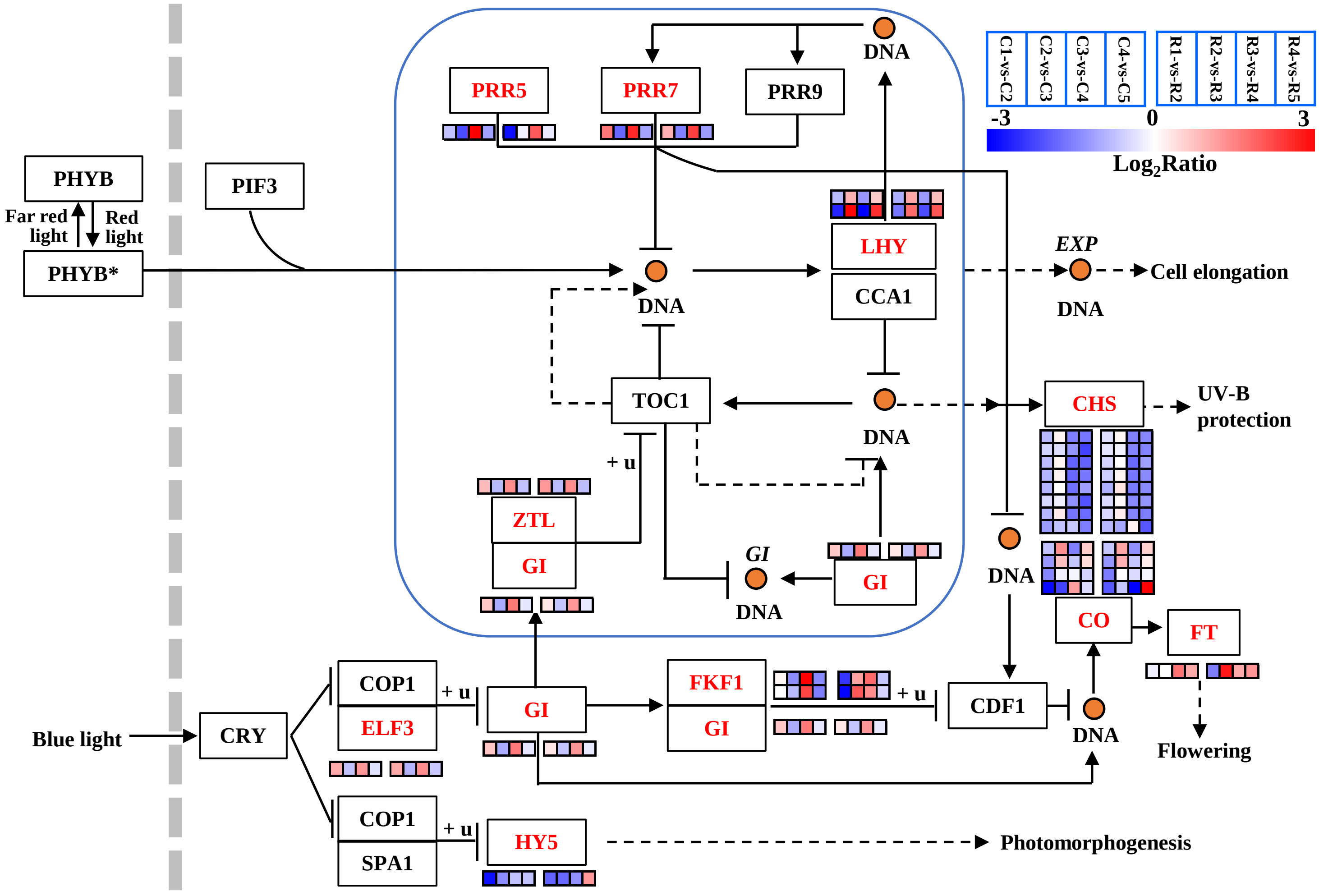

Figure S2 Significantly expressed genes are involved in the circadian rhythm-plant pathway during the flower bud's differentiation phases. Color codes from blue to red indicate changes in transcriptional expression levels between −3 and 3. The boxes from left to right indicate the expression values of a unigene obtained in C1 vs C2, C2 vs C3, C3 vs C4, C4 vs C5; R1 vs R2, R2 vs R3, R3 vs R4, R4 vs R5, respectively. Each row represents a separate unigene.

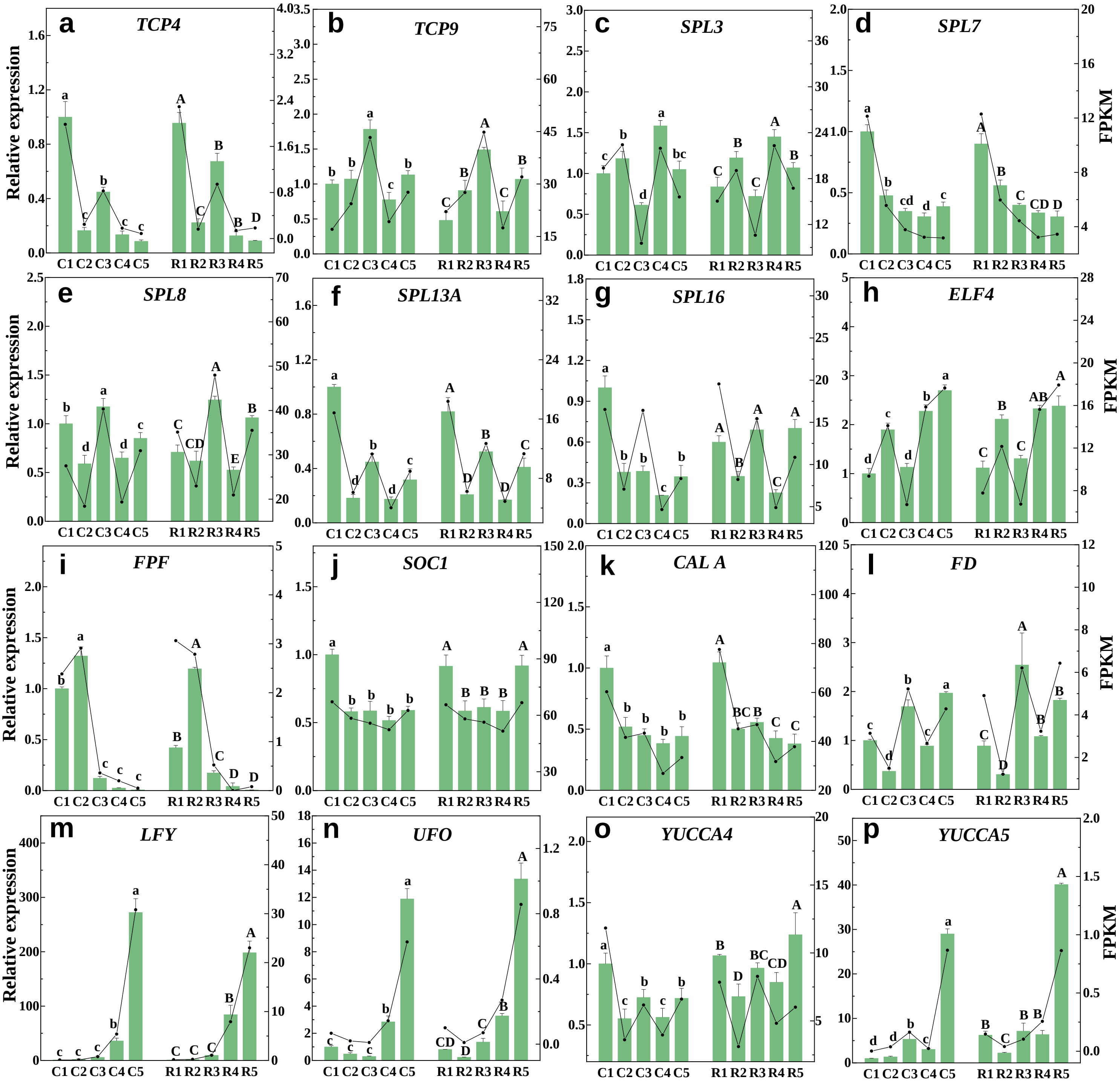

Figure S3 Expression of flowering related genes at different differentiation stages in two cultivation patterns, *TCP4* (a), *TCP9* (b), *SPL3* (c), *SPL7* (d), *SPL8* (e), *SPL13A* (f), *SPL16* (g), *ELF4* (h), *FPF* (i), *SOC1* (j), *CALA* (k), *FD* (l), *LFY* (m), *UFO* (n), *YUCCA4* (o), *YUCCA5* (p). The histogram and left axis are the relative expressions measured by qRT-PCR, and the dot-line and right axis are the FPKM value obtained by RNA-Seq. Error bars show the Mean ± SD of the three biological replicates. Different capital letters on the bars indicate significant differences between different stages of rain-sheltered model at $p < 0.05$ determined by Duncan's tests, lowercase letters indicate significant differences of shelter-free model.

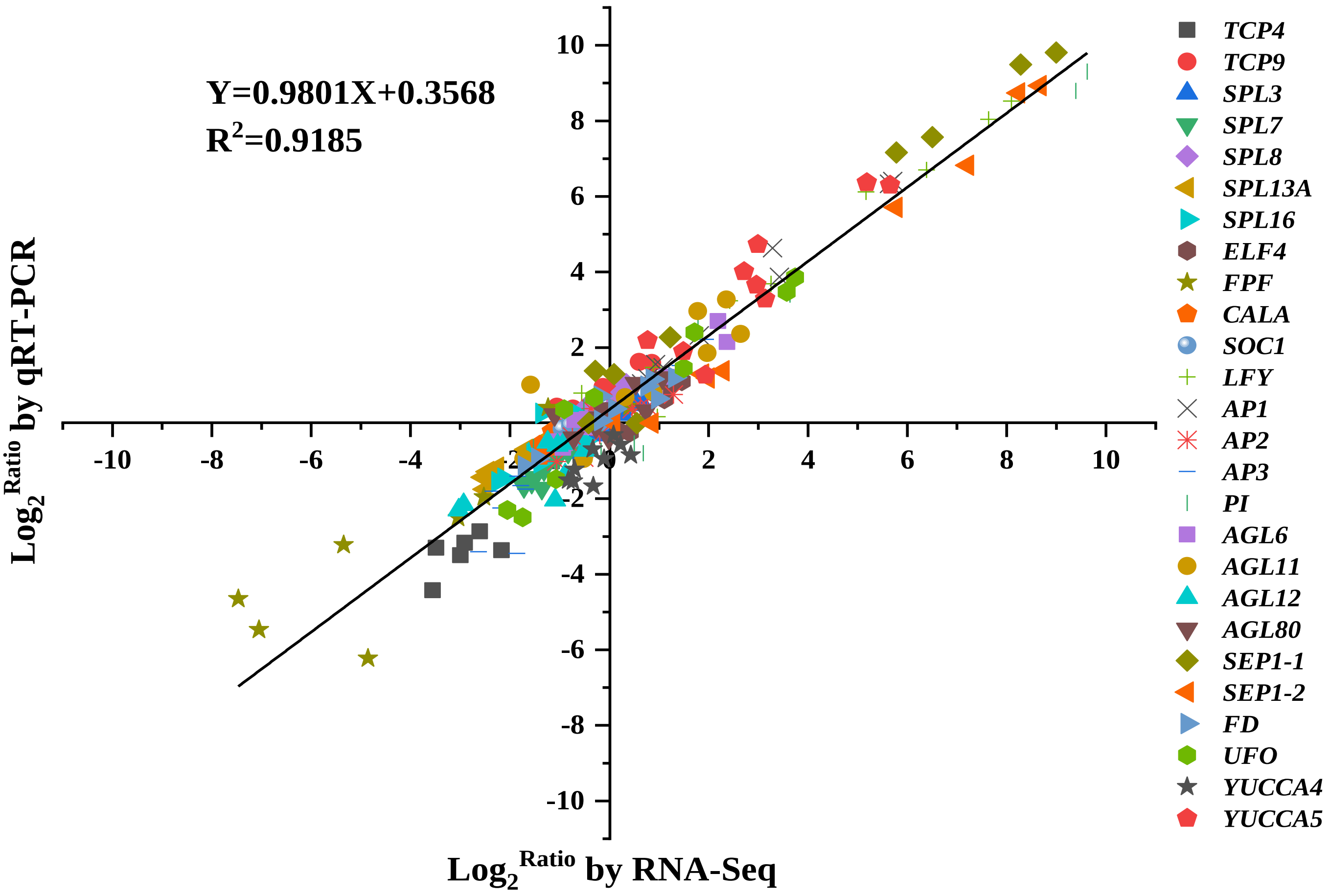

Figure S4 Correlation analysis between RNA-seq results and qRT-PCR data of ABCE model and flower bud differentiation genes.
